# Incidence of Complications for Different Approaches in Gynecomastia Correction: A Systematic Review of the Literature

**DOI:** 10.1007/s00266-022-02782-1

**Published:** 2022-02-09

**Authors:** Alessandro Innocenti, Dario Melita, Emanuela Dreassi

**Affiliations:** 1 Plastic and Reconstructive Microsurgery, Careggi University Hospital, Largo Palagi 1, 50127 Florence, Italy; 2 Department of Statistica, Informatica, Applicazioni (DiSIA), University of Florence, Florence, Italy

**Keywords:** Gynecomastia, Gynecomastia review, Surgical gland excision, Liposuction, Literature analysis

## Abstract

**Background:**

Gynecomastia is nowadays a very common disease, affecting a large cohort of patients with different ages. The aim of this literature review is to assess the incidence of complications with all proposed techniques and for combined procedures versus single approach procedures in gynecomastia correction.

**Materials and Methods:**

A systematic review of the literature was performed to identify all reported techniques for gynecomastia correction covering a period from January 1, 1987 to November 1, 2020. For all selected papers, demographic data, proposed technique, and complications’ incidence have been recorded.

**Results:**

A total number of 3970 results was obtained from database analysis. A final total number of 94 articles was obtained for 7294 patients analyzed. Patients have been divided into three groups: aspiration techniques, consisting in 874 patients (11,98%), surgical excision techniques, consisting in 2764 patients (37,90%), and combined techniques, consisting in 3656 patients (50,12%). Complications have been recorded for all groups, for a total number of 1407, of which 130 among “Aspiration techniques” group (14,87%), 847 among “Surgical excision techniques” group (30,64%), and 430 in “Combined techniques” group (11,76%).

**Conclusions:**

Several techniques have been proposed in the literature to address gynecomastia, with the potential to greatly improve self-confidence and overall appearance of affected patients. The combined use of surgical excision and aspiration techniques seems to reduce the rate of complications compared to surgical excision alone, but the lack of unique classification and the presence of several surgical techniques still represents a bias in the literature review.

**Level of Evidence III:**

This journal requires that authors assign a level of evidence to each article. For a full description of these Evidence-Based Medicine ratings, please refer to the Table of Contents or the online Instructions to Authors www.springer.com/00266.

## Introduction

Gynecomastia is defined as a benign enlargement of the mammary glands, commonly diffused among men. The prevalence of gynecomastia ranges from 38 to 64 percent in the male population [1].

Prevalence figures vary highly between age groups. Among male neonates, 60–90% have some amount of palpable breast tissue. The next chronological peak occurs during puberty with reported prevalence figures of 4–69% that decrease again by age 17 to approximately 10%. The third and last peak occurs in elderly men [2].

The etiology of gynecomastia is heterogeneous. More than 80% can be classified as idiopathic, since a well-established cause is not determined. Medical drugs, addictional drugs, and anabolic substance abuse, mostly among bodybuilders, have been identified as secondary causes for gynecomastia. The gynecomastia pathophysiology is due to a hormonal imbalance with decreased testosterone production, increased estrogen production, mainly from the peripheral conversion of androgens, and increased availability of estrogen precursors. In men, estrogen production results through aromatase activity to estradiol and estrone. In patients affected by gynecomastia, an increased local tissue sensitivity to estrogen metabolites is present [3].

Gynecomastia can affect normal self-esteem and sexual identity and often patients feel ashamed of their bodies during normal social activities.

Being a very popular item in the present literature, several surgical techniques have been proposed for gynecomastia correction. The aim of this systematic review is to assess the rate of reported complications with all proposed techniques and the evaluation of the complications’ rate in combined procedures versus single procedures.

## Material and Methods

### Literature Search

The searched databases included Medline, EMBASE, Cochrane and PubMed, covering a period from January 1, 1987 to November 1, 2020.

A detailed search was performed starting from the general topics to avoid overlooking the studies in the databases. Based on this, the keywords used for detailed investigation were “gynecomastia,” “gynecomastia surgery,” “gynecomastia correction,” “gynecomastia surgical correction”.

### Inclusion and Exclusion Criteria

Our predefined inclusion criteria included articles that included any age patients’ cohort, including pediatric population; included surgical techniques for the correction of gynecomastia (defined as any enlargement of the breast tissue); were English-language articles; were published between 1987 and 2020. Exclusion criteria were as follows: article considering non-surgical or therapeutic treatment for gynecomastia; articles about pseudogynecomastia; non-comparative studies, systematic reviews, case reports, expert opinions, conference and abstracts, review, letters to editors, and non-English articles.

### Data Extraction and Quality Assessment

**Fig. 1.**
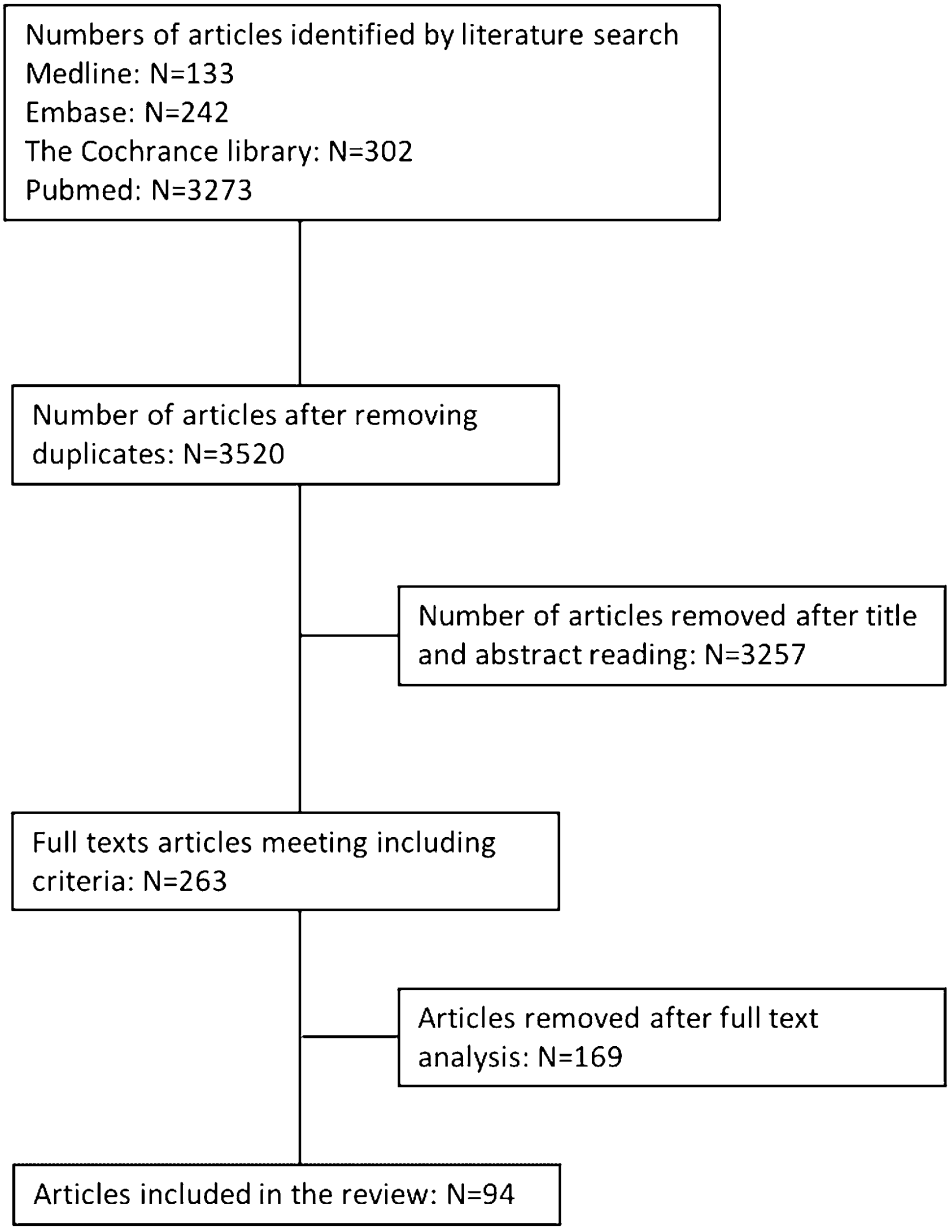
Flow chart for literature search

Two authors independently reviewed the titles and abstracts to assess eligibility for potential inclusion. The full-text papers were reviewed by two authors and inclusion was made on a consensus basis. Disagreement was resolved through a discussion between the reviewers. Literature analysis is reported in Fig. 1.

All articles have been separately analyzed for the following data:

1. Number of patients
2. Age range or, when the range was not indicated, mean age value
3. Proposed technique(s)
4. Complications

Since not all articles included patients’ satisfaction and gynecomastia’s grades, the authors decided not to collect those data to avoid bias.

The accurate analysis of all selected papers was conducted by both authors simultaneously. Proposed techniques have been categorized into three major groups according to their characteristics:

1. Aspiration, including techniques involving suction device(s), consisting in
2. Traditional liposuction
3. Ultrasound-assisted liposuction (UAL)
4. Suction-assisted liposuction (SAL)
5. Power-assisted liposuction (PAL)
6. Laser Lipolysis
7. Sharp cutting Liposuction
8. Mixed techniques
9. Surgical excision, including techniques with glandular removal, consisting in
10. Open excision
11. Endoscopically assisted surgical excision
12. Transaxillary excision
13. Microdebrider
14. Vacuum-assisted/Mammotome
15. Combined techniques, consisting in the combination of surgical excision and aspiration, including
16. Open excision and Liposuction/UAL/PAL
17. Pull-trough and Liposuction
18. Fragmentation and Liposuction
19. Cartilage shaver and Liposuction
20. Endoscopic adenectomy and Liposuction
21. Suction-Assisted excision and Liposuction

Complications have been statistically analyzed for all selected papers. In particular, the following complications have been recorded for each paper and grouped according to the proposed technique: hematoma, seroma, over-resection, under-resection, hypo- or hyperesthesia, wound dehiscence, infection, pathological scar, asymmetries, irregularities/redundant skin, NAC necrosis (partial or total)/abrasion and revision/recurrence.

### Statistical Analysis

For each study, the overall complication rate and the rate of each complication type was calculated. The complication rate across all studies, grouped according to the technique, was then calculated. Chi-square tests were used to compare complication rates between the groups. Data are shown in Table 1.

**Table 1:** Review of the literature for a single article, focusing on demographic parameters and complications rate

Authors	Patients	Age	Surgical technique	Complications												
				HE	SE	OR	UR	HH	WD	IN	PS	AS	IS	NN	RR	Total
Courtiss et al. [4]	101	16-61	SURGICAL EXCISION (hemiperiareolar)	31	18	36	42	21	0	0	36	0	0	0	0	184
	20		LIPOSUCTION	0	2	0	3	5	0	0	0	0	0	0	0	10
	38		SURGICAL EXCISION (hemiperiareolar)+ LIPOSUCTION	4	5	0	2	0	0	0	9	0	0	0	0	20
Aiache et al. [5]	38	NR	SURGICAL EXCISION (hemiperiareolar)	4	0	0	0	0	0	0	0	0	0	0	0	4
Ward et al. [6]	6	NR	SURGICAL EXCISION (horizontal ellipse with vertical pedicle)	1	0	0	0	0	0	0	0	0	0	0	0	1
Varma et al. [7]	20	23.5	SURGICAL EXCISION (hemiperiareolar)	2	1	0	0	0	0	0	0	0	0	0	0	3
Apesos et al. [8]	4	NR	LIPOSUCTION	0	0	0	1	0	0	0	0	0	0	0	0	1
	2		SURGICAL EXCISION (hemiperiareolar)+ LIPOSUCTION	0	0	0	0	0	0	0	0	0	0	0	0	0
Stark et al. [9]	14	16-34	LIPOSUCTION	0	0	0	0	0	0	0	0	0	0	0	0	0
	9		SURGICAL EXCISION + LIPOSUCTION	0	0	0	0	0	0	0	0	0	0	0	0	0
Brenner et al. [10]	44	NR	SURGICAL EXCISION (37 hemiperiareolar and 7 transverse)	0	0	0	0	2	0	0	4	0	0	0	0	6
Abramo et al. [11]	10	NR	SURGICAL EXCISION (hemiperiareolar)+ LIPOSUCTION	0	0	0	0	0	0	0	0	0	0	0	0	0
Samdal et al. [12]	3	16-69	SURGICAL EXCISION (hemiperiareolar)	1	0	0	0	0	0	0	0	0	0	0	0	1
	33		SURGICAL EXCISION (hemiperi- or circumareolar)+ LIPOSUCTION	2	0	2	1	0	0	0	0	0	0	0	0	5
	31		LIPOSUCTION	0	0	0	5	0	0	0	0	0	0	0	2	7
Morselli et al. [13]	11	NR	SURGICAL EXCISION (pull-trough) + LIPOSUCTION	0	0	0	0	0	0	0	0	0	0	0	0	0
Aiache et al. [14]	18	24-46	SURGICAL EXCISION (hemiperiareolar)+ LIPOSUCTION	0	0	0	0	0	0	0	0	0	0	0	0	0
Peters et al. [15]	11	13-18	SURGICAL EXCISION (bipedicled flap)	0	1	0	0	1	0	0	2	0	0	0	0	4
Hamas et al. [16]	31	12-67	SURGICAL EXCISION (hemiperiareolar)+ sharp cutting LIPOSUCTION	0	0	0	0	0	0	0	0	0	0	0	0	0
	57		Sharp cutting LIPOSUCTION	0	0	0	0	0	0	0	0	0	0	0	4	4
Smoot 3rd et al. [17]	20	NR	Purse-string SURGICAL EXCISION	0	0	0	0	0	0	0	0	0	0	0	2	2
Colombo-Benkmann et al. [18]	81	15-78	SURGICAL EXCISION (73 hemiperiareolar, 4 circumareolar, 4 submammary)	15	0	0	0	17	0	0	60	0	8	0	9	109
Gasperoni et al. [19]	64	16-62	SURGICAL EXCISION (hemiperiareolar)+ LIPOSUCTION	1	0	0	3	0	0	0	0	0	0	0	0	4
Javaid et al. [20]	4	NR	SURGICAL EXCISION (transareolar)	0	0	0	0	0	0	0	1	0	0	0	0	1
Babigian et al. [21]	2	NR	SURGICAL EXCISION (hemiperiareolar)	0	0	0	0	0	0	0	0	0	0	0	0	0
	18		SURGICAL EXCISION (hemiperiareolar)+ LIPOSUCTION	2	1	0	3	0	0	0	0	0	0	0	0	6
Persichetti et al. [22]	28	16-33	SURGICAL EXCISION (circumareolar)	0	1	0	0	0	2	0	0	0	0	0	0	3
Coskun et al. [23]	32	20-36	SURGICAL EXCISION (hemiperiareolar, in 10 cases extended)	7	0	0	0	1	0	0	9	0	3	1	0	21
Rohrich et al. [24]	61	NR	UAL or LIPOSUCTION	0	0	0	0	0	0	0	0	0	0	0	12	12
Boljanovic et al. [25]	3	NR	LIPOSUCTION	0	0	0	0	0	0	0	0	0	0	0	0	0
	25		SURGICAL EXCISION (hemiperiareolar)+ LIPOSUCTION	1	0	0	0	0	0	0	0	0	0	0	1	2
Fruhstorfer et al. [26]	31	13-57	31 UAL, SAL or LIPOSUCTION	0	0	0	3	1	0	0	0	0	0	2	1	7
	16		SURGICAL EXCISION + LIPOSUCTION	0	0	0	0	0	0	0	1	0	0	0	0	1
	1		SURGICAL EXCISION	0	0	0	0	0	0	0	0	0	0	0	0	0
Hammond et al. [27]	15	12-69	SURGICAL EXCISION (pull-through)+ LIPOSUCTION	0	1	0	0	1	0	0	1	0	0	0	0	3
Iwuagwu et al. [28]	5	16-88	SURGICAL EXCISION (mammotome)	0	0	0	0	0	0	0	0	0	0	0	0	0
Tashkandi et al. [29]	24	NR	SURGICAL EXCISION (purse-string)	0	0	0	0	0	0	0	0	0	0	0	0	0
Walden et al. [30]	12	25	LIPOSUCTION	0	0	0	0	0	0	0	0	0	0	0	0	0
	6		SURGICAL EXCISION (hemiperiareolar)	1	0	0	0	0	0	0	0	0	0	0	0	1
	16		SURGICAL EXCISION (hemiperiareolar)+ LIPOSUCTION	0	0	0	0	2	0	0	0	0	0	0	0	2
Gabra et al. [31]	39	9.5-17	SURGICAL EXCISION (circumareolar)	3	1	0	1	0	0	1	0	3	1	0	1	11
Bracaglia et al. [32]	45	21-65	SURGICAL EXCISION (pull-through) + LIPOSUCTION	2	0	0	1	0	0	0	0	0	0	0	1	4
Celebioglu et al. [33]	9	15-21	SURGICAL EXCISION (circumareolar with subareolar glandular pedicle)	0	0	0	0	9	0	0	1	0	0	1	1	12
Aslan et al. [34]	15	NR	SURGICAL EXCISION (periareolar–transareolar)	2	0	0	0	0	0	0	0	0	0	0	0	2
Prado et al. [35]	25	17-38	CARTILAGE SHAVER + LIPOSUCTION	0	0	0	0	0	0	0	0	0	0	0	0	0
Hodgson et al. [36]	31	16-57	UAL	0	0	0	0	0	0	0	0	0	1	0	1	2
Ramon et al. [37]	17	17-39	SURGICAL EXCISION (endoscopic pull-through) + LIPOSUCTION	0	0	0	0	0	0	0	0	0	0	0	0	0
Boni et al. [38]	38	23-64	LIPOSUCTION	0	0	0	0	0	0	0	0	0	0	0	0	0
Yavuz et al. [39]	5	18-24	Transaxillary SURGICAL EXCISION (Lighted Retractor-Assisted)	0	0	0	0	0	0	0	0	0	0	0	0	0
Haddad Filho et al. [40]	12	15-26	SURGICAL EXCISION (circumareolar)	0	0	0	0	0	0	0	0	0	0	0	0	0
Mentz et al. [41]	200	13-78	SURGICAL EXCISION (single puncture) + LIPOSUCTION	2	0	0	2	0	0	0	0	0	1	0	0	5
Esme et al. [42]	28	17-80	SURGICAL EXCISION (hemiperiareolar)+ LIPOSUCTION	0	0	0	0	0	0	0	0	0	0	0	0	0
Lista et al. [43]	96	17-46	SURGICAL EXCISION (pull-through) + LIPOSUCTION	0	2	0	0	0	0	0	0	0	0	0	0	2
Zhu et al. [44]	2	24-25	Endoscopically assisted SURGICAL EXCISION	0	0	0	0	0	0	0	0	0	0	0	0	0
Gheita et al. [45]	8	NR	SURGICAL EXCISION (Horizontal excision ellipse and superior pedicle flap)	0	0	0	0	0	0	0	0	0	0	0	0	0
Lanitis et al. [46]	102	11-82	SURGICAL EXCISION (56 circumareolar, 20 Inframammary fold, 10 concentric circumareolar, 12 inverted ‘‘T’’ reduction mastopexy, 4 extended circumareolar incision)	9	31	0	0	0	2	1	0	0	0	0	0	43
Cannistra et al. [47]	58	NR	SURGICAL EXCISION (Periareolar Incision and Dermal Double Areolar Pedicle) + SURGICAL EXCISION	0	0	0	0	6	0	0	0	0	0	0	0	6
Goh et al. [48]	8	NR	SURGICAL EXCISION (microdebrider)	0	0	0	0	0	0	0	1	0	0	1	0	2
Tu et al. [49]	22	13-63	SURGICAL EXCISION (periareolar zig-zag incision) + SURGICAL EXCISION	1	0	0	0	0	0	0	0	0	0	0	0	1
Scuderi et al. [50]	23	16-39	SURGICAL EXCISION (transareolar) + Power-assisted LIPOSUCTION	1	2	0	0	0	0	0	1	0	0	0	0	4
Fan et al. [51]	65	14-28	Endoscopically assisted SURGICAL EXCISION	0	1	0	0	0	0	0	0	0	0	2	0	3
Benito-Ruiz et al. [52]	40	19-57	CARTILAGE SHAVER + LIPOSUCTION	3	0	0	0	0	0	0	2	0	0	0	3	8
Rho et al. [53]	5	30-33	LASER LIPOLYSIS	0	0	0	0	0	0	0	0	0	0	0	0	0
Laituri et al. [54]	20	14-18	SURGICAL EXCISION (circumareolar or inferior pedicle reduction)	0	1	0	0	0	0	0	0	0	0	0	0	1
Petty et al. [55]	45	11-77	SURGICAL EXCISION	0	1	0	0	0	0	1	0	0	0	1	3	6
	56		SURGICAL EXCISION + LIPOSUCTION	2	6	0	0	0	0	0	0	0	0	1	3	12
	50		LIPOSUCTION	1	1	0	0	0	0	0	0	0	0	0	4	6
	76		CARTILAGE SHAVER + LIPOSUCTION	1	2	1	0	0	0	0	1	0	0	0	4	9
El Noamani et al. [56]	15	22-30	SURGICAL EXCISION (inferior pedicle without vertical scar)	0	0	0	0	0	1	0	3	0	0	1	0	5
Qutob et al. [57]	36	16-88	SURGICAL EXCISION (mammotome) + LIPOSUCTION	3	0	0	0	0	0	0	0	0	0	1	0	4
Cigna et al. [58]	37	18-43	SURGICAL EXCISION (hemiperiareolar) + Power-assisted LIPOSUCTION	1	0	0	0	0	0	0	0	0	0	0	0	1
He et al. [59]	20	18-47	SURGICAL EXCISION (mammotome)	1	0	0	0	0	0	0	0	0	0	0	0	1
Jarrar et al. [60]	1	18-44	Endoscopically assisted SURGICAL EXCISION	0	0	0	0	0	0	0	0	0	0	0	0	0
	7		Endoscopically assisted SURGICAL EXCISION + LIPOSUCTION	0	1	0	0	0	0	1	0	0	0	0	0	2
	4		LIPOSUCTION	0	0	0	0	0	0	0	0	0	0	0	0	0
Morselli et al. [61]	260	10-59	SURGICAL EXCISION (pull-through) + LIPOSUCTION	8	0	0	0	0	0	0	13	0	12	0	24	57
Trelles et al. [62]	28	24-56	LASER LIPOLYSIS	0	0	0	0	0	0	0	0	0	0	0	0	0
Zampieri et al. [63]	5	NR	SURGICAL EXCISION (circumareolar)	0	2	0	0	0	0	0	0	0	0	0	0	2
Lee et al. [64]	15	13-55	CARTILAGE SHAVER + LIPOSUCTION	1	0	0	0	0	0	0	0	3	0	0	0	4
Cao et al. [65]	58	17-52	Endoscopically assisted SURGICAL EXCISION	0	0	0	0	0	0	0	0	0	0	3	0	3
Hosnuter et al. [66]	23	15-42	SURGICAL EXCISION (superior periareolar) + LIPOSUCTION	0	0	0	0	0	1	0	0	0	0	0	0	1
Kasielska et al. [67]	113	17-54	SURGICAL EXCISION (94 circumareolar; 9 skin excision mastectomy; 6 inverted-T reduction mastopexy with NAC transposition; 4 inframammary fold approach with NAC graft )	8	4	0	0	11	0	1	0	0	0	1	0	25
Song et al. [68]	402	17-82	215 Periareolar incision, 97 complete concentric periareolar, 45 Inframammary fold incision, 26 Inverted-T incision, 53 Mammotome excision	7	10	0	0	2	0	0	10	0	0	9	6	44
	331	15-73	145 LIPOSUCTION, 241 UAL	4	7	0	0	26	0	0	0	0	0	0	2	39
Blau et al. [69]	1073	18-51	SURGICAL EXCISION (hemiperiareolar)	64	128	0	0	0	0	0	0	0	0	0	0	192
Yoo et al. [70]	13	20-28	1,444-nm Nd:YAG LAL	0	0	0	0	0	0	0	0	0	0	0	0	0
Schroder et al. [71]	53	13-66	SURGICAL EXCISION (hemiperiareolar)+ LIPOSUCTION	2	0	0	0	0	0	0	0	0	0	0	2	4
Ibrahiem et al. [72]	27	18-53	SURGICAL EXCISION (circumareolar with superior pedicle) + UAL	1	0	0	0	0	1	0	5	0	0	1	0	8
El-Sabbagh et al. [73]	18	13-33	SURGICAL EXCISION (hemiperiareolar)+ LIPOSUCTION	0	0	0	0	0	0	1	0	0	0	2	0	3
Shirol et al. [74]	20	16-36	SURGICAL EXCISION (orange pell hemiperiareolar)+ LIPOSUCTION	1	0	0	0	0	0	0	0	0	0	0	0	1
Bailey et al. [75]	75	NR	SURGICAL EXCISION (pull-through) + Power-assisted LIPOSUCTION	0	0	0	0	0	0	0	0	0	0	0	1	1
Kim et al. [76]	16	18-30	LIPOSUCTION	0	0	0	1	0	0	0	0	0	0	0	0	1
	48		SURGICAL EXCISION (hemi- or circumareolar)+ LIPOSUCTION	0	0	1	1	0	0	0	1	0	0	0	0	3
Innocenti et al. [77]	312	18-52	SURGICAL EXCISION (hemiperiareolar)+ LIPOSUCTION	4	6	0	0	0	0	0	0	0	47	0	3	60
Taheri et al. [78]	27	17-36	SURGICAL EXCISION (hemiperiareolar)+ LIPOSUCTION	0	0	0	0	9	0	0	1	1	0	4	0	15
Khalil et al. [79]	52	26.9	SURGICAL EXCISION (pull-through) + LIPOSUCTION	0	0	0	0	10	0	0	0	0	0	0	1	11
Sönmez Ergün et al. [80]	25	18-33	980 nm LASER LIPOLYSIS	0	2	0	0	0	0	0	0	4	0	0	0	6
Thienot et al. [81]	9	19-67	SURGICAL EXCISION (Postero-Inferior Pedicle) + LIPOSUCTION	1	0	0	0	0	1	0	1	0	0	0	0	3
Choi et al. [82]	71	16-18	SURGICAL EXCISION (hemiperiareolar)+ LIPOSUCTION	2	3	0	0	4	0	0	2	0	0	0	0	11
Ozalp et al. [83]	21	19-34	SAL	3	2	4	0	8	0	0	0	0	0	1	0	18
Lee et al. [84]	30	13-56	Cutting edge tip cannula + Power-assisted LIPOSUCTION	0	1	0	0	0	0	0	0	0	0	0	0	1
	10		SURGICAL EXCISION (hemiperiareolar)+ LIPOSUCTION	0	0	0	0	0	0	0	0	0	0	0	0	0
Wyrick et al. [85]	52	23-73	SURGICAL EXCISION (hemi- or circumareolar)+ LIPOSUCTION	2	4	0	0	0	0	0	0	0	0	0	0	6
Abdelrahman et al. [86]	18	28-34	LIPOSUCTION	0	0	0	2	0	0	1	0	0	0	0	0	3
Tarallo et al. [87]	15	18-28	SURGICAL EXCISION (hemiperiareolar)+ LIPOSUCTION	0	0	0	0	0	0	0	0	0	0	0	0	0
Yao et al. [88]	22	15-45	SURGICAL EXCISION (Vacuum-assisted)	1	0	0	0	1	0	0	0	0	1	0	0	3
Mohamad Hasan et al. [89]	150	NR	SURGICAL EXCISION (hemiperiareolar or Benelli)	40	29	0	0	24	8	0	2	0	0	15	0	118
Sim et al. [90]	101	26	SURGICAL EXCISION (microdebrider) + LIPOSUCTION	7	0	0	18	0	0	0	3	0	0	0	0	28
	31	27	LIPOSUCTION	3	0	0	10	0	0	0	0	0	0	0	0	13
	21	30	SURGICAL EXCISION (circumareolar)	4	0	0	6	0	0	0	1	0	0	0	0	11
	18	25	SURGICAL EXCISION (circumareolar) + LIPOSUCTION	4	0	0	4	0	0	0	1	0	0	0	0	9
Murugesan et al. [91]	149	19-57	SURGICAL EXCISION (pull-through) + LIPOSUCTION	2	0	0	0	0	0	0	0	0	0	0	0	2
Akhtar et al. [92]	30	17-38	SURGICAL EXCISION (hemiperiareolar)+ LIPOSUCTION	2	0	0	0	0	0	0	0	8	2	0	0	12
	30		SURGICAL EXCISION (arthroscopic shaver) + LIPOSUCTION	3	0	0	0	0	0	0	0	10	2	2	0	17
Tripathy et al. [93]	10	21-30	SURGICAL EXCISION (hemiperiareolar)+ LIPOSUCTION	2	0	0	0	0	0	0	0	0	0	0	0	2
	10		SURGICAL EXCISION (pull-through) + LIPOSUCTION	0	0	0	0	0	0	0	0	0	0	0	0	0
Harinatha et al. [94]	1159	NR	SURGICAL EXCISION (superior pedicle) + LIPOSUCTION	27	0	0	0	0	32	0	0	7	0	0	0	66
Jian et al. [95]	12	19-40	Endoscopically assisted SURGICAL EXCISION	0	0	0	0	1	0	0	0	0	0	0	0	1
Qu et al. [96]	56	NR	SURGICAL EXCISION (periareolar or inframammary fold)	1	0	0	0	5	0	0	0	0	0	0	0	6
	26		Vacuum-assisted SURGICAL EXCISION	3	0	0	0	2	0	0	0	0	0	0	0	5
Pfeiler et al. [97]	34	NR	SURGICAL EXCISION (hemiperiareolar)	8	1	0	0	0	0	2	0	0	0	0	0	11
	21		SURGICAL EXCISION (hemiperiareolar)+ LIPOSUCTION	3	1	0	0	0	0	1	0	0	0	0	0	5

*HE* hematoma(s); *SE* seroma; *OR* over-resection; *UR* under-resection; *HH* hypo-or hyperesthesia; *WD* wound dehiscence; *IN* infection; *PS* pathological scar; *AS* asymmetries; *IS* irregularities or redundant skin; *NN* NAC necrosis (partial or total) or abrasion; *RR* revision or recurrences

## Results

A total number of 3970 results was obtained from database analysis. A final total number of 94 articles was obtained, according to predefined inclusion and exclusion criteria, for a total number of 7294 patients analyzed [4–97].

Patients, according to previously mentioned criteria, have been divided into three groups:

- Aspiration techniques, consisting in 874 patients (11,98%)
- Surgical excision techniques, consisting in 2764 patients (37,90%)
- Combined techniques, consisting in 3656 patients (50,12%)

Among patients belonging to “Aspiration techniques” group, a further division into subgroups has been reported. Of these, 241 patients underwent traditional liposuction, 31 ultrasound-assisted liposuction, 21 suction-assisted liposuction, 71 laser lipolysis, 57 sharp cutting liposuction and 453 mixed techniques.

Among the 2764 patients belonging to “Surgical excision techniques” group, 2560 underwent traditional open excision, 138 endoscopically assisted adenectomy, 5 transaxillary excision, 8 microdebrider excision, and 73 vacuum-assisted/mammotome excision.

Of the 3656 patients belonging to “Combined techniques” group, 2396 underwent open excision and liposuction (either tradition, ultrasound-assisted or power-assisted), 713 pull-trough and liposuction, 301 excision by fragmentation and liposuction, 186 excision by cartilage shaver and liposuction, 24 endoscopic adenectomy and liposuction, and 36 suction-assisted excision and liposuction.

Complications have been recorded for all groups, for a total number of 1407, of which 130 among “Aspiration techniques” group (14,87%), 847 among “Surgical excision techniques” group (30,64%) and 430 in “Combined techniques” group (11,76%). Complications rate for each group is reported in Table 2. Most common complication recorded was hematoma (322 cases, 22,88%), mainly present in “surgical excision” techniques. This element could be addressed to the use of surgical excision alone in more severe forms, with a higher incidence of possible complications. For the same reasons, seroma rate is higher in “surgical excision” group.

**Table 2:** Complications rate for each group according to the inclusion criteria.

Technique	No.	Complications												
		Hematoma	Seroma	Over-resection	Under-resection	Hypo- or Hyperesthesia	Wound dehiscence	Infection	Pathological scar	Asymmetries	Irregularities or redundant skin	NAC necrosis (partial or total)/abrasion	Revision/ recurrence	Total
Aspiration	874	11	15	4	25	40	0	1	0	4	3	1	26	130
Traditional Liposuction	241	4	3	0	22	5	0	1	0	0	0	0	6	41
Ultrasound-Assisted Liposuction	31	0	0	0	0	0	0	0	0	0	1	0	1	2
Suction-Assisted Liposuction	21	3	2	4	0	8	0	0	0	0	0	1	0	18
Laser Lipolysis	71	0	2	0	0	0	0	0	0	4	0	0	0	6
Sharp cutting Liposuction	57	0	0	0	0	0	0	0	0	0	0	0	4	4
Mixed techniques	453	4	8	0	3	27	0	0	0	0	2	0	15	59
Surgical Exicision	2764	213	230	36	49	97	13	6	130	3	13	35	22	847
Open excision	2540	208	229	36	49	93	13	6	129	3	12	29	22	829
Endoscopically assisted	138	0	1	0	0	1	0	0	0	0	0	5	0	7
Transaxillary excision	5	0	0	0	0	0	0	0	0	0	0	0	0	0
Microdebrider	8	0	0	0	0	0	0	0	1	0	0	1	0	2
Vacuum-assisted/mammotome	73	5	0	0	0	3	0	0	0	0	1	0	0	9
Combined techniques	3656	98	34	4	35	32	35	3	42	29	64	16	38	430
Open excision and Liposuction/UAL/PAL	2396	66	28	3	14	21	35	2	22	16	49	8	9	273
Pull-trough and Liposuction	713	12	3	0	1	11	0	0	14	0	12	0	27	80
Fragmentation and Liposuction	301	9	0	0	20	0	0	0	3	0	1	0	0	33
Cartilage shaver and Liposuction	186	8	2	1	0	0	0	0	3	13	2	7	2	38
Endoscopic adenectomy and Liposuction	24	0	1	0	0	0	0	1	0	0	0	0	0	2
Suction-Assisted excision and Liposuction	36	3	0	0	0	0	0	0	0	0	0	1	0	4
Total	7294	322	279	44	109	169	48	10	172	36	80	52	86	1407
PERCENTAGE														
Aspiration	11,98	3,42	5,38	9,10	22,94	23,67	0,00	10,00	0,00	11,11	3,75	1,92	30,23	9,24
Surgical Exicision	37,90	66,15	82,44	81,81	44,95	57,40	27,08	60,00	75,58	8,33	16,25	67,31	25,58	60,20
Combined techniques	50,12	30,43	12,18	9,09	32,11	18,93	72,92	30,00	24,42	80,56	80,00	30,77	44,19	30,56

*UAL* ultrasound-assisted liposuction; *PAL* power-assisted liposuction.

**Fig. 2.**
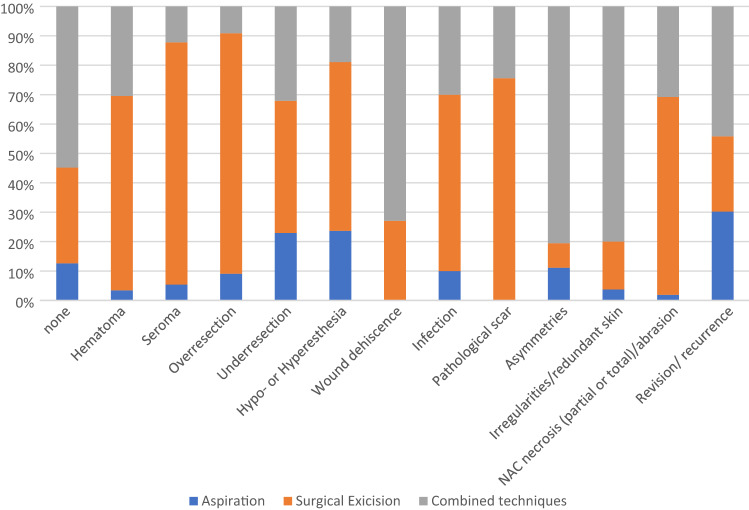
Percentages distribution of patients subjected to a technique for each outcome

**Fig. 3.**
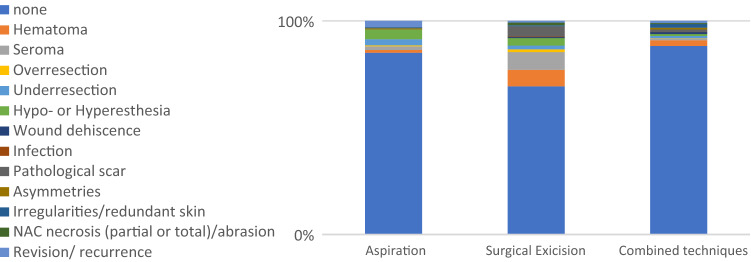
Graphic representation of percentages distribution of patient’s outcome for each technique

From statistical descriptive analysis, we observe that using different techniques we obtain different percentages of patients with no complications and with the considered complications (Figs. 2 and 3).

Follow a statistical inference approach, we test, using Pearson's Chi-squared test, the null hypothesis of independence between technique and outcome; we observe a value of 760,49 for the test statistic with 24 degrees of freedom, with a very small *p*-value (*p*-value < 2.2e−16). This suggests us to reject the null hypothesis, confirming that different techniques give different outcomes.

## Discussion

Several techniques have been described throughout the years for treating gynecomastia. Aspiration techniques, including liposuction and its modern variations, base their principles on removing trough a minimal access to the redundant fatty and breast tissues by fragmentation and suction. Since gynecomastia in most cases is defined as mixed, aspiration of the gland cannot permit histopathological analysis and skin redistribution is limited. Moreover, these techniques do not permit a direct hemostasis [98–101].

Aspiration techniques vary according to the modality used for fat and glandular tissue removal. In suction-assisted liposuction, after tumescent solution infiltration, localized areas of unwanted fat are removed through the combination of a high-vacuum blunt-tipped cannula and longitudinal motion. In ultrasound-assisted liposuction, ultrasound frequencies produced by specific cannulas primarily affect tissues with the lowest density, such as fat tissues, whose density is further reduced by previous wetting with tumescent solution. Interactions between adipose tissue and ultrasound waves lead to adipocyte fragmentation trough cavitation and, therefore, this technique has a high degree of selectivity for fat cells resulting in a high degree of selectivity for fat cells, and thus reducing blood loss, postoperative edema, and ecchymosis and avoiding contour irregularities. In power-assisted liposuction, oscillating rotational and translational movements of cannula tip are produced, mimicking the motion of the operator’s arm with lower amplitude and allowing an easier penetration of fibrous fat and glandular tissue, while generating no thermal energy and therefore reducing the risk of cutaneous burns. Laser lipolysis utilizes the principles of selective photothermolysis to preferentially lyse adipocytes while leaving surrounding structures unaffected. Different laser wavelengths may vary in their relative effectiveness in targeting substances present in the subcutaneous environment. Thus, lasers achieve their desired effect via photolysis of adipose cells, photocoagulation of small vessels, liberation of adipocyte lipases, and contraction of dermal collagen.

More challenging cases, such as male tuberous breast, can hardly be corrected only with aspiration techniques since an open excision is required to manage the deformity [102–105].

Open excision techniques base their principle on a direct view and management of the gland, through several types of surgical accesses according to the surgeon’s preference and entity of the defect [106, 107]. The main advantage of open excision is the direct control of the hemostasis and redundant skin control, with the main disadvantage of permanent scars, whose quality cannot be predicted. Furthermore, gland excision can permit histopathological analysis since male breast carcinoma, even if rare, can occur only in patients affected by gynecomastia [108].

Combined techniques are usually composed of an open excision phase followed by an aspiration phase: the combination of these techniques can permit a limited scar extension since, after open excision, the wide undermining of the skin flap onto a larger area can often permit a sufficient skin redistribution [109–112].

Since gynecomastia represents a disease commonly diffused worldwide, an updated systematic review that focuses not only on the different types of proposed treatment but also on complications rate, is a useful tool for plastic surgeons [113]. Several biases can be found, mostly related to the high variations in proposed treatments and clinical classifications. In fact, several articles proposed specific treatments for graded gynecomastia patients, but the large variations of gynecomastia classifications cannot guarantee a statistical comparison and therefore only the type of surgical approach, despite the grade of the disease, have been considered [114–116]. Moreover, no comparison of patients’ postoperative satisfaction has been performed because of the absence of evaluation in some papers and for the different used methods for evaluation [117–121]. Besides those biases, that are strictly relative to the large discussion on this topic in literature, this review, as previously stated, confirms that the combined approach with traditional surgical excision of glandular tissue combined with liposuction provides the lowest rate of complications, compared to aspiration techniques alone and surgical excision techniques alone [4–97]. As an adjunctive element for discussion, authors retain that, despite its rare incidence, breast cancer in male affected by gynecomastia can occur, and therefore, histopathological analysis is mandatory and can be performed only with surgical excision rather than with aspiration techniques [122, 123]. Since psychological assessments have been largely discussed in literature, this aspect, even if fundamental, have not been included in this review. Focusing on surgical treatment, articles including medical treatment for gynecomastia have been excluded from this review. This review evidences the need for a single classification method, including also minor forms, and for a validated and universal method for the evaluation of satisfaction [124]. In this review, the male tuberous breast has not been included. Even if it presents peculiar clinical hallmarks, it is still poorly investigated in literature and often misdiagnosed with other forms of gynecomastia [125]. A general consensus on this condition, and its inclusion in gynecomastia classification, will help plastic surgeons in the diagnosis and management of this condition. To avoid bias, also pseudogynecomastia, due to massive weight loss, has not been included since its treatment and rate of complications differ from gynecomastia surgery [126, 127]. We personally retain that the higher incidence of complications among patients who underwent surgical excision is strictly related to the high number of patients and to the fact that these techniques are often used to treat the most severe forms, compared to aspiration techniques and combined techniques [128]. Moreover, surgical excision techniques have been early described in the literature, and the evolution of techniques has reduced the complications rate.

## Conclusion

Several techniques have been proposed in the literature to address gynecomastia, with the potential to greatly improve the self-confidence and overall appearance of affected patients. The combined use of surgical excision and aspiration techniques seems to reduce the rate of complications compared to surgical excision alone, but lack of unique classification and the presence of several surgical techniques still represents a bias in the literature review.

## References

[1] Fruhstorfer BH, Malata CM (2003). A systematic approach to the surgical treatment of gynaecomastia. Br J Plast Surg.

[2] Cuhaci N, Polat SB, Evranos B, Ersoy R, Cakir B (2014). Gynecomastia: Clinical evaluation and management. Indian J Endocrinol Metab.

[3] Wollina U, Goldman A (2011). Minimally invasive esthetic procedures of the male breast. J Cosmet Dermatol.

[4] Courtiss EH (1987). Gynecomastia: analysis of 159 patients and current recommendations for treatment. Plast Reconstr Surg.

[5] Aiache AE (1989). Surgical treatment of gynecomastia in the body builder. Plast Reconstr Surg.

[6] Ward CM, Khalid K (1989). Surgical treatment of grade III gynaecomastia. Ann R Coll Surg Engl.

[7] Varma SK, Henderson HP (1990). A prospective trial of adrenaline infiltration for controlling bleeding during surgery for gynaecomastia. Br J Plast Surg.

[8] Apesos J, Chami R (1991). Functional applications of suction-assisted lipectomy: a new treatment for old disorders. Aesth Plast Surg.

[9] Stark GB, Grandel S, Spilker G (1992). Tissue suction of the male and female breast. Aesth Plast Surg.

[10] Brenner P, Berger A, Schneider W, Axmann HD (1992). Male reduction mammoplasty in serious gynecomastias. Aesth Plast Surg.

[11] Abramo AC (1994). Axillary approach for gynecomastia liposuction. Aesth Plast Surg.

[12] Samdal F, Kleppe G, Amland PF, Abyholm F (1994). Surgical treatment of gynaecomastia. Five years' experience with liposuction. Scand J Plast Reconstr Surg Hand Surg.

[13] Morselli PG (1996). "Pull-through": a new technique for breast reduction in gynecomastia. Plast Reconstr Surg.

[14] Aiache AE (1998). Secondary surgery for failed gynecomastia correction from liposuction. Aesthet Surg J.

[15] Peters MH, Vastine V, Knox L, Morgan RF (1998). Treatment of adolescent gynecomastia using a bipedicle technique. Ann Plast Surg.

[16] Hamas RS, Williams CW (1998). A sharp cutting liposuction cannula for gynecomastia. Aesthet Surg J.

[17] Smoot EC (1998). Eccentric skin resection and purse-string closure for skin reduction with mastectomy for gynecomastia. Ann Plast Surg.

[18] Colombo-Benkmann M, Buse B, Stern J, Herfarth C (1999). Indications for and results of surgical therapy for male gynecomastia. Am J Surg.

[19] Gasperoni C, Salgarello M, Gasperoni P (2000). Technical refinements in the surgical treatment of gynecomastia. Ann Plast Surg.

[20] Javaid M, Shibu M (2000). Surgical correction of gynaecomastia: a new approach. Br J Plast Surg.

[21] Babigian A, Silverman RT (2001). Management of gynecomastia due to use of anabolic steroids in bodybuilders. Plast Reconstr Surg.

[22] Persichetti P, Berloco M, Casadei RM, Marangi GF, Di Lella F, Nobili AM (2001). Gynecomastia and the complete circumareolar approach in the surgical management of skin redundancy. Plast Reconstr Surg.

[23] Coskun A, Duzgun SA, Bozer M, Akinci OF, Uzunkoy A (2001). Modified technique for correction of gynaecomastia. Eur J Surg.

[24] Rohrich RJ, Ha RY, Kenkel JM, Adams WP (2003). Classification and management of gynecomastia: defining the role of ultrasound-assisted liposuction. Plast Reconstr Surg.

[25] Boljanovic S, Axelsson CK, Elberg JJ (2003). Surgical treatment of gynecomastia: liposuction combined with subcutaneous mastectomy. Scand J Surg.

[26] Fruhstorfer BH, Malata CM (2003). A systematic approach to the surgical treatment of gynaecomastia. Br J Plast Surg.

[27] Hammond DC, Arnold JF, Simon AM, Capraro PA (2003). Combined use of ultrasonic liposuction with the pull-through technique for the treatment of gynecomastia. Plast Reconstr Surg.

[28] Iwuagwu OC, Calvey TA, Ilsley D, Drew PJ (2004). Ultrasound guided minimally invasive breast surgery (UMIBS): a superior technique for gynecomastia. Ann Plast Surg.

[29] Tashkandi M, Al-Qattan MM, Hassanain JM, Hawary MB, Sultan M (2004). The surgical management of high-grade gynecomastia. Ann Plast Surg.

[30] Walden JL, Schmid RP, Blackwell SJ (2004). Cross-chest lipoplasty and surgical excision for gynecomastia: a 10-year experience. Aesthet Surg J.

[31] Gabra HO, Morabito A, Bianchi A, Bowen J (2004). Gynaecomastia in the adolescent: a surgically relevant condition. Eur J Pediatr Surg.

[32] Bracaglia R, Fortunato R, Gentileschi S, Seccia A, Farallo E (2004). Our experience with the so-called pull-through technique combined with liposuction for management of gynecomastia. Ann Plast Surg.

[33] Celebioğlu S, Ertaş NM, Ozdil K, Oktem F (2004). Gynecomastia treatment with subareolar glandular pedicle. Aesthetic Plast Surg.

[34] Aslan G, Tuncali D, Terzioglu A, Bingul F (2005). Periareolar-transareolar-perithelial incision for the surgical treatment of gynecomastia. Ann Plast Surg.

[35] Prado AC, Castillo PF (2005). Minimal surgical access to treat gynecomastia with the use of a power-assisted arthroscopic-endoscopic cartilage shaver. Plast Reconstr Surg.

[36] Hodgson EL, Fruhstorfer BH, Malata CM (2005). Ultrasonic liposuction in the treatment of gynecomastia. Plast Reconstr Surg.

[37] Ramon Y, Fodor L, Peled IJ, Eldor L, Egozi D, Ullmann Y (2005). Multimodality gynecomastia repair by cross-chest power-assisted superficial liposuction combined with endoscopic-assisted pull-through excision. Ann Plast Surg.

[38] Boni R (2006). Tumescent power liposuction in the treatment of the enlarged male breast. Dermatology.

[39] Yavuz M, Kesiktas E, Kesiktas NN, Acartürk S (2006). Lighted retractor-assisted transaxillary approach in gynecomastia correction. Ann Plast Surg.

[40] Haddad Filho D, Arruda RG, Alonso N (2006). Treatment of severe gynecomastia (Grade III) by resection of periareolar skin. Aesthet Surg J.

[41] Mentz HA, Ruiz-Razura A, Newall G, Patronella CK, Miniel LA (2007). Correction of gynecomastia through a single puncture incision. Aesthet Plast Surg.

[42] Esme DL, Beekman WH, Hage JJ, Nipshagen MD (2007). Combined use of ultrasonic-assisted liposuction and semicircular periareolar incision for the treatment of gynecomastia. Ann Plast Surg.

[43] Lista F, Ahmad J (2008). Power-assisted liposuction and the pull-through technique for the treatment of gynecomastia. Plast Reconstr Surg.

[44] Zhu J, Huang J (2008). Surgical management of gynecomastia under endoscope. J Laparoendosc Adv Surg Tech A.

[45] Gheita A (2008). Gynecomastia: the horizontal ellipse method for its correction. Aesthet Plast Surg.

[46] Lanitis S, Starren E, Read J, Heymann T, Tekkis P, Hadjiminas DJ, Al Mufti R (2008). Surgical management of Gynaecomastia: outcomes from our experience. Breast.

[47] Cannistra C, Piedimonte A, Albonico F (2009). Surgical treatment of gynecomastia with severe ptosis: periareolar incision and dermal double areolar pedicle technique. Aesthet Plast Surg.

[48] Goh T, Tan BK (2010). Song C (2010) Use of the microdebrider for treatment of fibrous gynaecomastia. J Plast Reconstr Aesthet Surg.

[49] Tu LC, Tung KY, Chen HC, Huang WC, Hsiao HT (2009). Eccentric mastectomy and zigzag periareolar incision for gynecomastia. Aesthet Plast Surg.

[50] Scuderi N, Dessy LA, Tempesta M, Bistoni G, Mazzocchi M (2010). Combined use of power-assisted liposuction and trans-areolar incision for gynaecomastia treatment. J Plast Reconstr Aesthet Surg.

[51] Fan L, Yang X, Zhang Y, Jiang J (2009). Endoscopic subcutaneous mastectomy for the treatment of gynecomastia: a report of 65 cases. Surg Laparosc Endosc Percutan Tech.

[52] Benito-Ruiz J, Raigosa M, Manzano M, Salvador L (2009). Assessment of a suction-assisted cartilage shaver plus liposuction for the treatment of gynecomastia. Aesthet Surg J.

[53] Rho YK, Kim BJ, Kim MN, Kang KS, Han HJ (2009). Laser lipolysis with pulsed 1064 nm Nd:YAG laser for the treatment of gynecomastia. Int J Dermatol.

[54] Laituri CA, Garey CL, Ostlie DJ, St Peter SD, Gittes GK, Snyder CL (2010). Treatment of adolescent gynecomastia. J Pediatr Surg.

[55] Petty PM, Solomon M, Buchel EW, Tran NV (2010). Gynecomastia: evolving paradigm of management and comparison of techniques. Plast Reconstr Surg.

[56] El Noamani S, Thabet AM, Enab AA, Shaeer O, El-Sadat A (2010). High grade gynecomastia: surgical correction and potential impact on erectile function. J Sex Med.

[57] Qutob O, Elahi B, Garimella V, Ihsan N, Drew PJ (2010). Minimally invasive excision of gynaecomastia- a novel and effective surgical technique. Ann R Coll Surg Engl.

[58] Cigna E, Tarallo M, Fino P, De Santo L, Scuderi N (2011). Surgical correction of gynecomastia in thin patients. Aesthet Plast Surg.

[59] He Q, Zheng L, Zhuang D, Fan Z, Xi C, Zhou P (2011). Surgical treatment of gynecomastia by vacuum-assisted biopsy device. J Laparoendosc Adv Surg Tech A.

[60] Jarrar G, Peel A, Fahmy R, Deol H, Salih V, Mostafa A (2011). Single incision endoscopic surgery for gynaecomastia. J Plast Reconstr Aesthet Surg.

[61] Morselli PG, Morellini A (2012). Breast reshaping in gynecomastia by the "pull-through technique": considerations after 15 years. Eur J Plast Surg.

[62] Trelles MA, Mordon SR, Bonanad E, Moreno Moraga J, Heckmann A, Unglaub F, Betrouni N, Leclère FM (2013). Laser-assisted lipolysis in the treatment of gynecomastia: a prospective study in 28 patients. Lasers Med Sci.

[63] Zampieri N, Castellani R, Modena S, Camoglio FS (2012). Class III gynecomastia in pediatric age: a new modified surgical treatment. Pediatr Surg Int.

[64] Lee JH, Kim IK, Kim TG, Kim YH (2012). Surgical correction of gynecomastia with minimal scarring. Aesthet Plast Surg.

[65] Cao H, Yang ZX, Sun YH, Wu HR, Jiang GQ (2013). Endoscopic subcutaneous mastectomy: a novel and effective treatment for gynecomastia. Exp Ther Med.

[66] Hoşnuter M (2014). An ameliorated approach for sharp resection in gynecomastia surgery. Indian J Surg.

[67] Kasielska A, Antoszewski B (2013). Surgical management of gynecomastia: an outcome analysis. Ann Plast Surg.

[68] Song YN, Wang YB, Huang R, He XG, Zhang JF, Zhang GQ, Ren YL, Pang JH, Pang D (2014). Surgical treatment of gynecomastia: mastectomy compared to liposuction technique. Ann Plast Surg.

[69] Blau M, Hazani R (2015). Correction of gynecomastia in body builders and patients with good physique. Plast Reconstr Surg.

[70] Yoo KH, Bae JM, Won CY, Chung YS, Goo B, Rho YK, Kim GM, Lee J, Ahn BH, Kim BJ (2015). Laser-assisted liposuction using the novel 1444-nm Nd:YAG laser for the treatment of gynecomastia: a pilot study. Dermatology.

[71] Schröder L, Rudlowski C, Walgenbach-Brünagel G, Leutner C, Kuhn W, Walgenbach KJ (2015). Surgical strategies in the treatment of gynecomastia grade I-II: the combination of liposuction and subcutaneous mastectomy provides excellent patient outcome and satisfaction. Breast Care (Basel).

[72] Ibrahiem SM (2016). Severe gynecomastia: new technique using superior pedicle NAC flap through a circumareolar approach. Ann Plast Surg.

[73] El-Sabbagh AH (2016). Combined approach for gynecomastia. GMS Interdiscip Plast Reconstr Surg DGPW.

[74] Shirol SS (2016). Orange peel excision of gland: a novel surgical technique for treatment of gynecomastia. Ann Plast Surg.

[75] Bailey SH, Guenther D, Constantine F, Rohrich RJ (2016). Gynecomastia management: an evolution and refinement in technique at UT Southwestern medical center. Plast Reconstr Surg Glob Open.

[76] Kim DH, Byun IH, Lee WJ, Rah DK, Kim JY, Lee DW (2016). Surgical management of gynecomastia: subcutaneous mastectomy and liposuction. Aesthetic Plast Surg.

[77] Innocenti A, Melita D, Mori F, Ciancio F, Innocenti M (2017). Management of gynecomastia in patients with different body types: considerations on 312 consecutive treated cases. Ann Plast Surg.

[78] Taheri AR, Farahvash MR, Fathi HR, Ghanbarzadeh K, Faridniya B (2016). The satisfaction rate among patients and surgeons after periareolar surgical approach to gynecomastia along with liposuction. World J Plast Surg.

[79] Khalil AA, Ibrahim A, Afifi AM (2017). No-drain single incision liposuction pull-through technique for gynecomastia. Aesthetic Plast Surg.

[80] Sönmez Ergün S, Kayan RB, Güleş ME, Kuzu İM (2017). Effects of laser-assisted lipolysis on nipple-areola complex. J Cosmet Laser Ther.

[81] Thiénot S, Bertheuil N, Carloni R, Méal C, Aillet S, Herlin C, Watier E (2017). Postero-inferior pedicle surgical technique for the treatment of grade III gynecomastia. Aesthet Plast Surg.

[82] Choi BS, Lee SR, Byun GY, Hwang SB, Koo BH (2017). The characteristics and short-term surgical outcomes of adolescent gynecomastia. Aesthet Plast Surg.

[83] Özalp B, Berköz Ö, Aydınol M (2018). Is the transposition of the nipple-areolar complex necessary in Simon grade 2b gynecomastia operations using suction-assisted liposuction?. J Plast Surg Hand Surg.

[84] Lee YK, Lee JH, Kang SY (2018). Gynecomastia: glandular-liposculpture through a single transaxillary one hole incision. J Plast Surg Hand Surg.

[85] Wyrick DL, Roberts M, Young ZT, Mancino AT (2018). Changing practices: the addition of a novel surgical approach to gynecomastia. Am J Surg.

[86] Abdelrahman I, Steinvall I, Mossaad B, Sjoberg F, Elmasry M (2018). Evaluation of glandular liposculpture as a single treatment for grades I and II gynaecomastia. Aesthet Plast Surg.

[87] Tarallo M, Di Taranto G, Fallico N, Ribuffo D (2019). The round-the-clock technique for correction of gynecomastia. Arch Plast Surg.

[88] Yao Y, Yang Y, Liu J, Wang Y, Zhao Y (2019). Vacuum-assisted minimally invasive surgery: an innovative method for the operative treatment of gynecomastia. Surgery.

[89] Mohamad Hasan R (2019). Modified Benelli procedure for subcutaneous mastectomy in gynecomastia: a randomised controlled trial. Ann Med Surg (Lond).

[90] Sim N, Tan G, Tan BK, Goh T (2020). Review of the microdebrider excision and liposuction technique (MELT) for the treatment of gynecomastia. J Plast Reconstr Aesthet Surg.

[91] Murugesan L, Karidis A (2020). External quilting: new technique to avoid haematoma in gynaecomastia surgery. Aesthetic Plast Surg.

[92] Akhtar A, Eitezaz F, Rashid M, Khan I, Malik SA (2019). Liposuction in gynecomastia: an assessment of the suction-assisted arthroscopic shaver versus open disc excision techniques. Cureus.

[93] Tripathy S, Likhyani A, Sharma R, Sharma RK (2020). Prospective analysis and comparison of periareolar excision (delivery) technique and pull-through technique for the treatment of gynecomastia. Aesthet Plast Surg.

[94] Harinatha S (2020). Male gynecomastia correction by superior dynamic flap method: a consistent and versatile technique. World J Plast Surg.

[95] Jian C, Wu L, Lin L, Liu W, Zheng Z, Yang C (2020). Single-port endoscopic mastectomy via the lateral chest approach for the treatment of grade II gynecomastia. Med (Baltim).

[96] Qu S, Zhang W, Li S, He W, Lu R, Zhang Q, Zhang J, Wang N (2021). The vacuum-assisted breast biopsy system is an effective strategy for the treatment of gynecomastia. Aesthet Plast Surg.

[97] Pfeiler PP, Luketina R, Dastagir K, Vogt PM, Mett TR, Kaltenborn A, Könneker S (2021). Expected reduction of the nipple-areolar complex over time after treatment of gynecomastia with ultrasound-assisted liposuction mastectomy compared to subcutaneous mastectomy alone. Aesthet Plast Surg.

[98] Innocenti A, Mori F, Melita D, Innocenti M, Ciancio F (2018). Discussion on "reduction of the areolar diameter after ultrasound-assisted liposuction for gynecomastia". Ann Plast Surg.

[99] Innocenti A, Melita D, Innocenti M (2018). Re: a novel method to insert drain atraumatically after liposuction in gynecomastia. Indian J Plast Surg..

[100] Innocenti A, Melita D (2021). The vacuum-assisted breast biopsy system is an effective strategy for the treatment of gynecomastia. Aesthet Plast Surg.

[101] Innocenti A, Melita D (2021). Aesthetic outcome of gynecomastia management with conventional liposuction and cross-chest liposuction: a prospective comparative study. Aesthet Plast Surg.

[102] Innocenti A, Serena G, Innocenti M (2021). External quilting: new technique to avoid haematoma in gynaecomastia surgery. Aesthet Plast Surg.

[103] Innocenti A (2019). Male tuberous breast: a rare variant of gynecomastia. Clinical considerations and personal experience: tips and tricks to maximize surgical outcomes. Aesthet Plast Surg.

[104] Innocenti A, Ghezzi S, Innocenti M (2019). Correction of tuberous nipple areolar complex deformity in gynecomastia: the deformity that can get forgotten. Ann Plast Surg.

[105] Innocenti A, Melita D (2021). Percutaneous intradermal purse-string closure for correction of male tuberous nipple-areola complex deformity. Aesthet Plast Surg.

[106] Innocenti A, Ciancio F, Parisi D, Portincasa A, Melita D, Innocenti M (2017). Comment to "orange peel excision of gland: a novel surgical technique for treatment of gynecomastia". Ann Plast Surg.

[107] Innocenti A, Ghezzi S, Melita D (2020) Commentary on “surgical treatment of gynaecomastia: a standard of care in plastic surgery” by Tobias R. Mett, Peter P. Pfeiler, Rosalia Luketina, Alperen S. Bingöl, Nicco Krezdorn & Peter M. Vogt. Eur J Plast Surg 43(5): 1-2

[108] Innocenti A, Ghezzi S, Melita D, Ciancio F, Innocenti,  (2018). Comment to: "complications and outcomes after gynecomastia surgery: analysis of 204 pediatric and 1583 adult cases from a national multi-center database". Aesthet Plast Surg.

[109] Innocenti A, Ciancio F, Portincasa A, Parisi D (2017). Discussion: surgical management of gynecomastia-subcutaneous mastectomy and liposuction. Aesthet Plast Surg.

[110] Innocenti A, Melita D (2021). Expected reduction of the nipple-areolar complex over time after treatment of gynecomastia with ultrasound-assisted liposuction mastectomy compared to subcutaneous mastectomy alone. Aesthet Plast Surg.

[111] Innocenti A, Melita D, Mori F, Ciancio F, Innocenti M (2017). Reply to the comment on: management of gynecomastia in patients with different body types: considerations on 312 consecutive treated cases. Ann Plast Surg.

[112] Innocenti A, Ghezzi S, Melita D, Innocenti M (2018). Clinical characteristics of asymmetric bilateral gynecomastia: suggestion of desirable surgical method based on a single-institution experience. Aesthet Plast Surg.

[113] Innocenti A, Melita D, Ciancio F, Innocenti M (2017). Discussion: "long-term follow-up of recurrence and patient satisfaction after surgical treatment of gynecomastia". Aesthet Plast Surg.

[114] Innocenti A, Melita D, Mori F, Ciancio F, Innocenti M (2017). Comment to "postero-inferior pedicle surgical technique for the treatment of grade III gynecomastia". Aesthet Plast Surg.

[115] Innocenti A, Melita D, Innocenti M (2018). Evaluation of glandular liposculpture as a single treatment for grades I and II gynecomastia. Aesthet Plast Surg.

[116] Innocenti A, Melita D, Ghezzi S (2019). Closed-suction drains after subcutaneous mastectomy for gynecomastia: do they reduce complications?. Aesthet Plast Surg.

[117] Ciancio F, Innocenti A, Parisi D, Portincasa A (2017). Gynecomastia -classification for surgical management: a systematic review and novel classification system. Plast Reconstr Surg.

[118] Innocenti A, Ghezzi S, Melita D, Innocenti M (2017). Comment on: "surgical masculinization of the breast: clinical classification and surgical procedures". Aesthet Plast Surg.

[119] Innocenti A, Melita D, Ghezzi S, Ciancio F, Innocenti M (2017). Comment to: "the characteristics and short-term surgical outcomes of adolescent gynecomastia". Aesthet Plast Surg.

[120] Innocenti A, Melita D (2021). Patients decision-making characteristics affects gynecomastia treatment satisfaction: a multicenter study using the BODY-Q chest module. Aesthet Plast Surg.

[121] Innocenti A, Melita D (2021). Endoscopic axillary approach improves patient satisfaction of gynecomastia subcutaneous mastectomy: a cross-sectional study using the BODY-Q chest module. Aesthet Plast Surg.

[122] Innocenti A, Melita D, Mori F, Innocenti M, Ciancio F (2018). Discussion on "gynecomastia surgery-impact on life quality: a prospective case-control study". Ann Plast Surg.

[123] Melita D, Innocenti A (2020). Prospective analysis and comparison of periareolar excision (delivery) and pull-through technique for the treatment of gynecomastia. Aesthet Plast Surg.

[124] Fagerlund A, Lewin R, Rufolo G, Elander A, Santanelli di Pompeo F, Selvaggi G (2015). Gynecomastia: a systematic review. J Plast Surg Hand Surg.

[125] Innocenti A, Melita D, Innocenti M (2021). Gynecomastia and chest masculinization: an updated comprehensive reconstructive algorithm. Aesthet Plast Surg.

[126] Barone M, Cogliandro A, Tsangaris E, Salzillo R, Morelli Coppola M, Ciarrocchi S, Brunetti B, Tenna S, Tambone V, Persichetti P (2018). Treatment of severe gynecomastia after massive weight loss: analysis of long-term outcomes measured with the italian version of the BODY-Q. Aesthet Plast Surg.

[127] Barone M, Cogliandro A, Persichetti P (2019). Innovative technique to improve chest shape following gynecomastia correction in post-bariatric surgery patients. Plast Reconstr Surg.

[128] Innocenti A, Ciancio F, Francesco M, Melita D, Innocenti M (2017). Comment to "no-drain single incision liposuction pull-through technique for gynecomastia". Aesthet Plast Surg.

